# Early and strong antibody responses to SARS-CoV-2 predict disease severity in COVID-19 patients

**DOI:** 10.1186/s12967-022-03382-y

**Published:** 2022-04-15

**Authors:** Jānis Plūme, Artis Galvanovskis, Sindija Šmite, Nadezhda Romanchikova, Pawel Zayakin, Aija Linē

**Affiliations:** 1 Cancer Biomarker Group, Latvian Biomedical Research and Study Centre, Ratsupites Str 1, k-1, Riga, 1067 Latvia; 2 Faculty of Biology, University of Latvia, Riga, Latvia

**Keywords:** SARS-CoV-2, Antibody profile, Antigen microarray, Prognosis, IgG, IgA, IgE, Mast cells

## Abstract

**Background:**

Antibody response to SARS-CoV-2 is a valuable biomarker for the assessment of the spread of the virus in a population and evaluation of the vaccine candidates. Recent data suggest that antibody levels also may have a prognostic significance in COVID-19. Most of the serological studies so far rely on testing antibodies against spike (S) or nucleocapsid (N) protein, however antibodies can be directed against other structural and nonstructural proteins of the virus, whereas their frequency, biological and clinical significance is unknown.

**Methods:**

A novel antigen array comprising 30 SARS-CoV-2 antigens or their fragments was developed and used to examine IgG, IgA, IgE and IgM responses to SARS-CoV-2 in sera from 103 patients with COVID-19 including 34 patients for whom sequential samples were available, and 20 pre-pandemic healthy controls.

**Results:**

Antibody responses to various antigens are highly correlated and the frequencies and peak levels of antibodies are higher in patients with severe/moderate disease than in those with mild disease. This finding supports the idea that antibodies against SARS-CoV-2 may exacerbate the severity of the disease via antibody-dependent enhancement. Moreover, early IgG and IgA responses to full length S protein may be used as an additional biomarker for the identification of patients who are at risk of developing severe disease. Importantly, this is the first study reporting that SARS-CoV-2 elicits IgE responses and their serum levels positively correlate with the severity of the disease thus suggesting a link between high levels of antibodies and mast cell activation.

**Conclusions:**

This is the first study assessing the prevalence and dynamics IgG, IgA, IgE and IgM responses to multiple SARS-CoV-2 antigens simultaneously. Results provide important insights into the pathogenesis of COVID-19 and have implications in planning and interpreting antibody-based epidemiological studies.

**Supplementary Information:**

The online version contains supplementary material available at 10.1186/s12967-022-03382-y.

## Introduction

Most patients with COVID-19 develop antibody responses against SARS-CoV-2 antigens. Serological tests for the detection of anti-SARS-CoV-2 antibodies appear to have a limited value for diagnosing a current infection, whereas it is the main instrument for the epidemiological studies, accurate assessment of the infection fatality risk, evaluating immune responses against vaccine candidates and identifying suitable convalescent plasma donors. Moreover, they may turn out to be valuable tools for the management of patients, however there still are important knowledge gaps regarding the heterogeneity, prognostic value, kinetics and biological roles of anti-SARS-CoV-2 antibody responses.

The antibodies can be directed against the structural proteins of SARS-CoV-2 virus: Spike (S), nucleocapsid (N), envelope (E) and membrane (M) protein, as well as against various nonstructural proteins, including ORF3b and ORF8 [1]. Antibodies against the receptor-binding domain (RBD) and S1 and S2 subunits of S protein have been shown to block viral attachment and/or entry into the host cell thereby neutralizing the infection [2, 3], whereas the biological and clinical significance of antibodies against the other antigens is unknown. Serological tests that are currently in use detect IgG, IgA or IgM-class antibodies or pan-Ig against full-length S or N proteins or their fragments [4–6], therefore most of the studies have assessed antibody responses to one or two SARS-CoV-2 antigens, but the landscape of antibody responses against various antigens is poorly characterized.

In the current study, we developed an antigen array comprising 30 SARS-CoV-2 antigens: various forms of full-length S, N and M proteins and S subunits, fragments of NSP1-5, ORF3b, ORF8 and 11 epitopes predicted within the S protein. The antigen array was tested for reactivity with IgG, IgA, IgM and IgE antibodies in sera from 103 patients with COVID-19 including 31 patients for whom sequential samples were available, and 20 pre-pandemic healthy controls. To the best of our knowledge, this is the first study assessing the antibody responses to multiple SARS-CoV-2 antigens simultaneously and measuring serum IgE levels in patients with COVID-19. Results allowed assessment of prevalence and dynamics of antibody responses to various antigens and revealed a correlation between the antibody levels and severity of the disease.

## Materials and methods

### Serum samples

Serum samples from 103 patients with PCR-confirmed COVID-19 and 20 age-matched pre-pandemic controls were obtained from the Latvian Genome Database. Fifty five from the enrolled patients have been hospitalized at various hospitals in Latvia between April 2020 and January 2021. Longitudinal samples were collected from 31 patients at 2–4 time points during hospitalization or routine check-up in the clinic for recovered patients. The number of samples collected at various time points is shown in Table 1. Blood samples from the patients who have not been hospitalized were collected at the COVID-19 testing labs and the clinical information about the symptoms of the disease was collected using a standardized questionnaire. The pre-pandemic controls (10 males and 10 females) were enrolled in the biobank before 2019. The study was approved by the Central Committee of Medical Ethics of Latvia (decision No. 01-29.1.2/928). The blood samples were collected after the patients’ informed written consent was obtained.

**Table 1:** Number of patients and samples collected at various time points

Blood draws	Number of patients	Number of samples per timepoint		
		1–14 DPS	15–84 DPS	> 84 DPS
Single	72	27	31	14
Multiple	31	24	41	10

The disease severity was defined according to the guidelines by Latvian Ministry of Health as follows: mild disease is characterized by fever, malaise, cough, upper respiratory symptoms, and/or less common features of COVID-19, in the absence of dyspnea; moderate disease—pneumonia with dyspnea, infiltrates on chest imaging, in the absence of hypoxia and need for oxygenation; severe disease—clinical signs of pneumonia plus one of the following: blood oxygen saturation < 94%, respiratory failure and need for oxygenation or ventilatory support, and respiratory rate > 30 breaths/min.

The blood samples were collected in venous blood collection tubes with clot activator (Becton Dickinson, US), centrifuged at 4000 RPM for 15 min, aliquoted and stored at − 80 °C until use.

### Prediction of epitopes

Linear B cell epitopes were predicted from the SARS-CoV-2 genome (NC_045512.2) translation by analyzing S, N, M, E, NSP1-5, ORF3b and ORF8 protein sequences using BepiPred-2.0, ABCpred and SVMTriP tools as described before [7, 8]. BepiPred-2.0 threshold was increased from 0.5 to 0.6 to increase specificity at the cost of sensitivity. Location of the predicted epitopes in the 3D protein structures was analyzed using the online 3D viewing tool Mol* 3D Viewer from RCSB PDB and only those epitopes that were located on the surface were selected for the study. Some closely spaced epitopes on continuous protein structures were combined into one expression unit. Details about the selected epitopes are provided in Additional file 1: Table S1.

### Production of recombinant proteins

The codon optimized DNA sequences encoding the predicted epitopes were synthesized and inserted into pGEX-6P-3 vector by BioCat GmbH (Germany). The DNA sequences were inserted at the 3’end of the GST tag and a His-tag was introduced at the 3’end of the insert. The recombinant proteins were expressed in *E. coli* BL21-DE3 cells and purified from cell culture lysates using GST-tagged columns according to manufacturer's protocol (GST SpinTrap; GE Healthcare UK Limited). The quality of proteins was checked by Western blot using anti-His tag antibody (6x-His Tag Monoclonal Antibody (HIS.H8), Invitrogen).

### Production and processing of antigen array

Recombinant full length SARS-CoV-2 proteins or protein subunits were purchased from BioCat GmbH (Germany). Details about the commercial proteins are provided in Additional file 1: Table S2. Peptides encoding the predicted epitopes were expressed in house as described above. All proteins were diluted to 0.2 mg/mL in phosphate-buffered saline (PBS) buffer and printed in triplicates on nitrocellulose-coated 16-pad FAST slides (GVS Filter Technology, Italy) using QArray Mini microarray printer (Genetix, UK). Human serum albumin (P1493-200, BioVision, USA) and glutathione-containing protein elution buffer were used as negative controls. The slides were processed as described previously with some modifications [9]. Briefly, the slides were dried and blocked in 7% (w/v) milk powder in TBS, 0.05% Tween-20. For the detection of IgG, IgA and IgM-class antibodies, the slides were incubated with 1:200 diluted human serum samples for 2 h, washed four times in TBS with 0.5% Tween 20 for 15 min and then incubated with 1:1500 diluted secondary antibodies: Alexa Fluor® 647 AffiniPure Goat Anti-Human IgG (109-605-098, Jackson ImmunoResearch, USA), Alexa Fluor® 647 AffiniPure Goat Anti-Human IgA (109-605-011, Jackson ImmunoResearch, USA) and Alexa Fluor® 647 AffiniPure Rabbit Anti-Human IgM (309–605-095, Jackson ImmunoResearch, USA). For the detection of IgE-class antibodies, 1:5 diluted sera and 1:500 diluted mouse anti- human IgE monoclonal antibody I27 Alexa Fluor® 647 (ENZ-ABS224-0100, Enzo Life Sciences, Inc., USA) was used.

### Data processing and statistical analysis

The arrays were scanned at 10 µm resolution in PowerScanner (Tecan, Switzerland). The results were recorded as TIFF files and the raw data were processed with GenePix™ Pro 4.0 software (Molecular Devices, USA) and further analyzed using an ad hoc program composed in R as described previously [9, 10]. Briefly, the mean Cy5 signals were background subtracted, averaged between replicates and normalized to the negative control spots. The cut-off value for defining reactive antigens in each array was set at median plus 2 SD of pre-pandemic control samples.

The Mann–Whitney U test was used to compare antibody levels between two independent groups of samples. The two-tailed Spearman correlation analysis was used to evaluate the relationship between antibody responses to various antigens. Receiver operating characteristic (ROC) curve analysis was used to estimate the performance of antibody levels for classification of patients with severe and moderate *vs* mild disease.

## Results

### Generation and performance of the SARS-CoV-2 antigen array

The antigen array comprised the following SARS-CoV-2 proteins or protein fragments: commercial full-length S protein (S_FL_), super stable S trimer (S_FL3_), RBD (S_RBD_), S1 (S_S1sub_) and S2 subunits (S_S2sub_), N (N_FL_) and M (M_FL_) proteins, as well as 23 in-house produced protein fragments representing predicted epitopes within the S, M and N proteins, NSP1-5, ORF3b and ORF8 (Additional file 1). Human serum albumin and glutathione-containing elution buffers served as negative controls, while human serum IgG, IgA and IgM served as positive controls. A representative image of testing the antigen array with serum from COVID-19 patient is shown in Additional file 2.

Inter-assay reproducibility was assessed by testing the antigen array with two serum samples in three replicates on different days. The mean coefficient of variability (CV) for IgG antibodies was 0.204 and for IgA—0.439 that is acceptable variability for immunoassays. Linear dynamic range of the IgG assay was assessed by testing the array with serial dilutions (1:100; 1:400; 1:1600 and 1:6400) of 4 serum samples. An example of the results is shown in Additional file 2. Results showed that the signal intensities for the majority of antigens were within the linear dynamic range for the whole range of dilutions, except the strongest signals that reached the saturation at 1:100 dilution. Serum dilution of 1:200 was chosen for the detection of IgG, IgA and IgM responses in order to reach the maximum sensitivity while maintaining the dynamic range of the assay. IgE is the least abundant antibody class constituting only approximately 0.05% of the serum immunoglobulins [11], therefore the detection of IgE responses to SARS-CoV-2 required lower dilutions of serum and secondary antibodies—1:5 and 1:500, respectively.

### Dynamics of antibody responses to various SARS-CoV-2 antigens

Dynamics of antibody responses against various SARS-CoV-2 antigens was studied in 75 longitudinal samples from 31 COVID-19 patients, including 24 samples that were collected ≤ 14 days after the onset of symptoms (DPS).

**Fig. 1:**
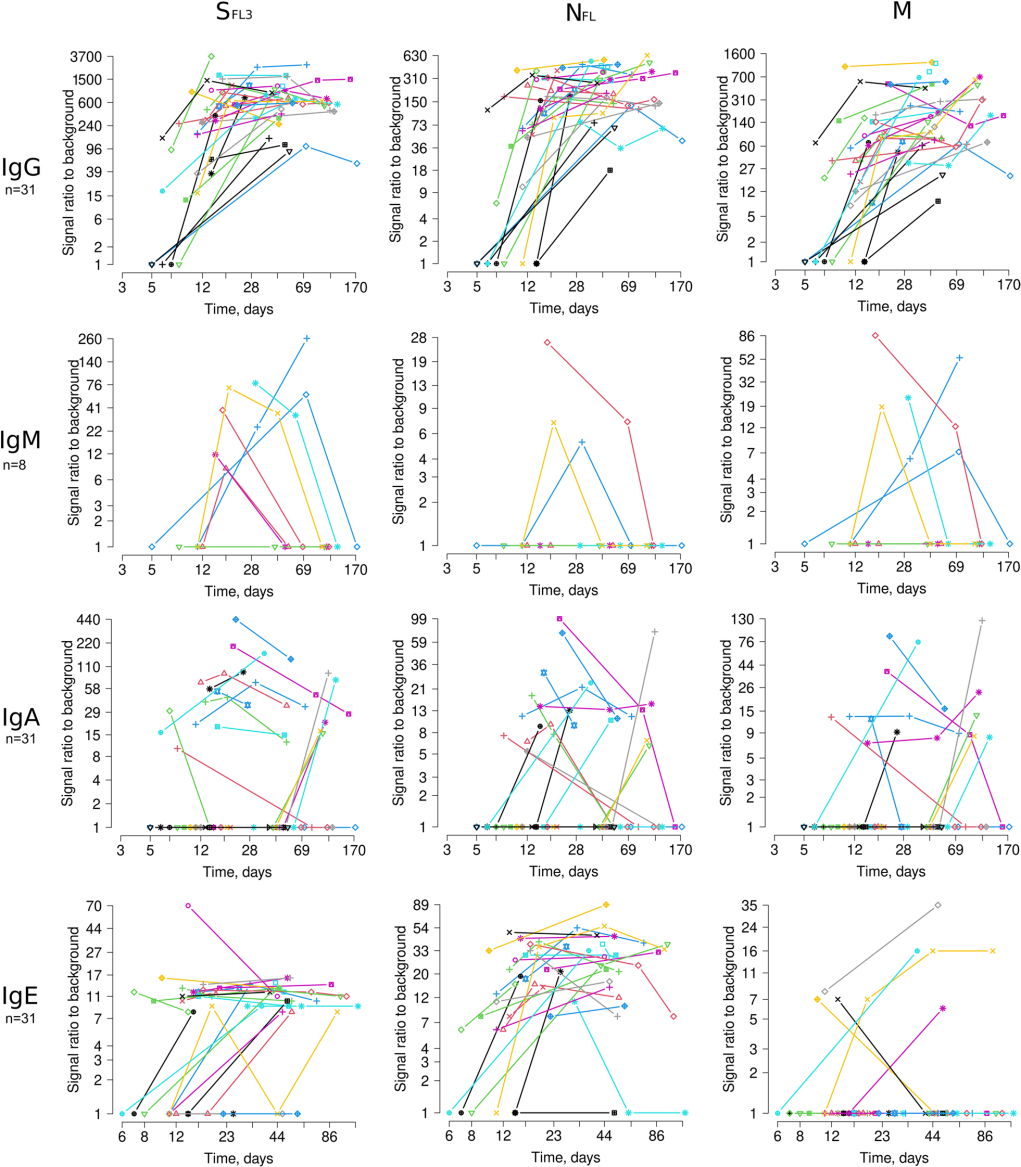
Dynamics of antibody responses against SARS-CoV-2 S_FL3_, N_FL_ and M antigens in a longitudinal cohort of 31 COVID-19 patients. IgM responses were tested in serial samples from 8 patients

IgGs against S_FL3_, S_FL_, S_RBD_, S_S1sub_ and S_S2sub_, M_FL_ and N_FL_ were already present in 66.7 to 81.5% of the samples collected before 14 DPS (S_FL3_, N_FL_ and M_FL_ are shown in Fig. 1). The remaining patients became seropositive in the second time point (days 15–70). In line with the previous studies [12, 13], the IgG levels reached the plateau between days 12 and 20 and stayed relatively stable at least over 3 months. The timing of seroconversion for the other antigens was ranging from day 5 to 59. However, the frequency and mean signal intensity was also significantly lower; hence the later seroconversion may be related to higher limit of detection of these antibodies, not later antigen exposure or slower response of B cells.

IgM responses were tested in 8 patients. As expected, IgM levels against S_FL3_, S_FL_, S_RBD_, S_S1sub_ and S_S2sub_, M_FL_ and N_FL_ were lower than those for IgG and were rapidly decreased starting from day 18 in all except one case. This suggested that the IgM levels do not have a plateau phase and are not comparable between the groups of samples collected at various time-points, therefore the IgM responses were not tested in the remaining samples.

**Fig. 2:**
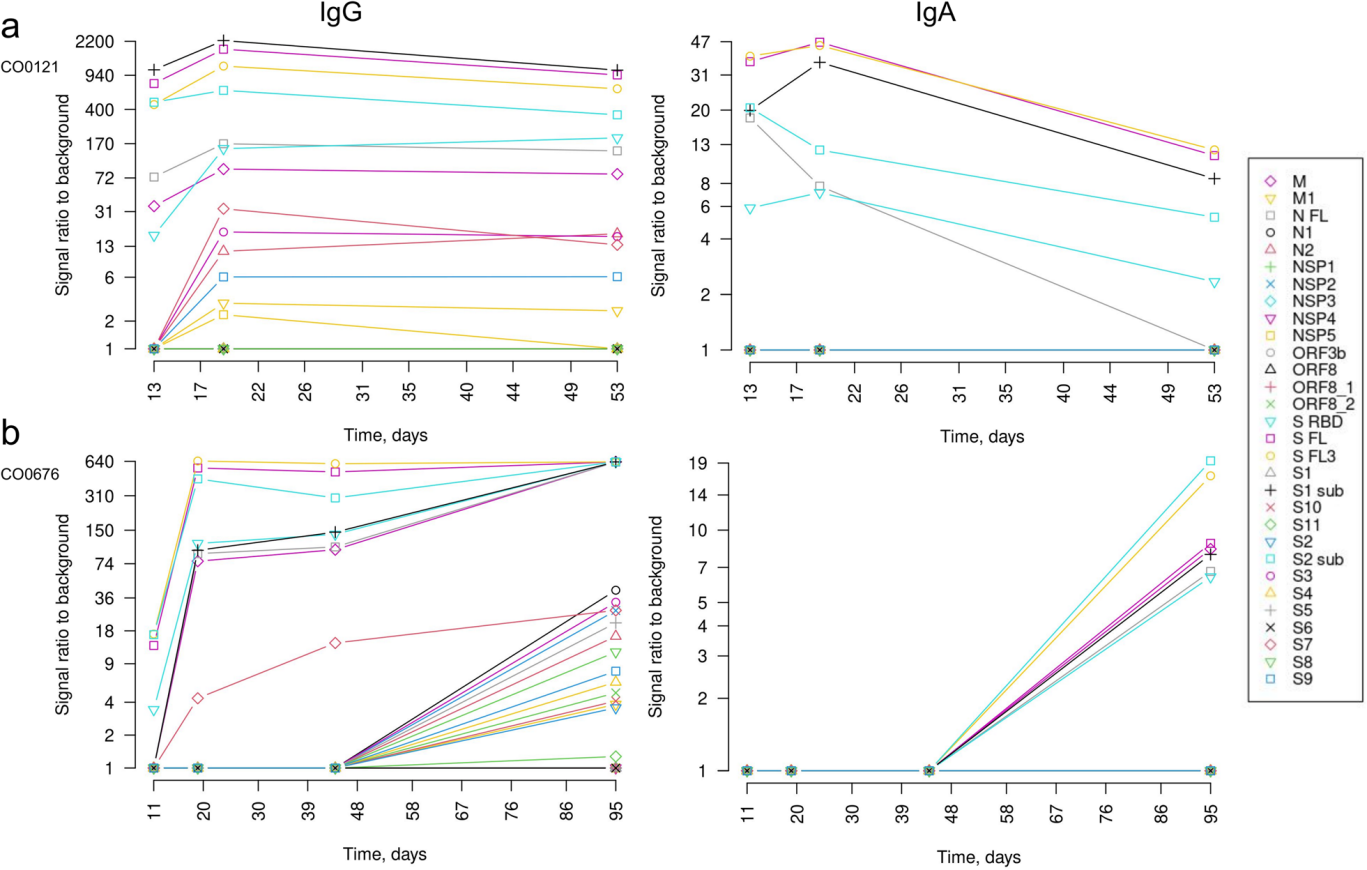
Antibody profiles in two COVID-19 patients. **a** IgG and IgA profile in a patient with early synchronized IgG and IgA response. **b** IgG and IgA profile in a patient with delayed IgA response

The kinetics of IgA responses was more heterogeneous among the patients. Fourteen patients had IgA responses against various forms of S, N and M proteins in samples collected before 14 DPS and the levels tended to decrease in the subsequent time-points. In these patients, the IgA seroconversion appears to occur simultaneously with the IgG seroconversion (IgG and IgA profiles of a representative case are shown in Fig. 2a). Whereas 6 patients were seronegative for IgAs against all or some of the antigens until 40–65 DPS and seroconverted before the subsequent blood draw (95–120 DPS). A representative case of delayed IgA response is shown in Fig. 2b. Interestingly, in these patients, IgG responses against the S, N and M proteins were present in the earliest time points but the seroconversion of IgG against a number of individual epitopes and non-structural proteins occurred before the last blood draw. Such pattern of seroconversion suggests that despite the activation of B cells by the day 20, epitope spreading and Ig class switching may occur at much later time point in a fraction of patients. Although all these patients had fully recovered and had no clinical signs of the disease between the third and fourth blood draw, we cannot exclude that the delayed seroconversion is triggered by the re-infection.

The timing of seroconversion of IgE responses against N_FL_ was comparable with that of IgG responses and the anti-N_FL_ IgE levels remained relatively stable over the time course of this study. Anti-S_FL3_ IgEs seroconverted later, however it is possible that the signal reached the detection limit only once it was close to the peak level. IgEs against other antigens were found in a substantially smaller fraction of patients and the assessment of seroconversion was not meaningful.

### Prevalence of IgG, IgA and IgE antibodies against various SARS-CoV-2 antigens

The prevalence of IgG, IgA and IgE antibodies against various SARS-CoV-2 antigens was analyzed in serum samples collected from 73 COVID-19 patients > 14 DPS (Table 2), when the majority of patients were expected to be seroconverted. In cases where multiple serum samples were available, the sample collected between 15 and 85 DPS was selected for this analysis.

**Table 2:** Frequencies of IgG, IgA and IgE responses in sera of COVID-19 patients collected > 14 DPS, %

Antigen	Mild, %			Moderate, %			Severe, %			Total, %		
	IgG	IgA	IgE	IgG	IgA	IgE	IgG	IgA	IgE	IgG	IgA	IgE
Full-lenght proteins or subunits												
S_FL3_	94.7	16.7	66.7	100	38.5	92.3	100	54.5	90	97.3	30.1	79.7
S_FL_	97.4	25	12.1	100	42.3	50	100	54.5	60	98.7	35.6	33.3
S_RBD_	92.1	22.2	0	100	42.3	3.85	100	63.6	10	96	35.6	2.9
S_1sub_	89.5	22.2	0	100	42.3	34.6	100	63.6	20	94.7	35.6	15.9
S_2sub_	100	25	3.03	100	42.3	19.2	100	54.5	20	100	35.6	11.6
N_FL_	94.7	19.4	66.7	100	46.2	96.2	100	45.5	100	97.3	32.9	82.6
M_FL_	84.2	13.9	0	100	30.8	23.1	100	45.5	0	92	24.7	8.7
Predicted epitopes or protein fragments												
S1	5.26	0	0	7.69	0	0	27.3	0	0	9.33	0	0
S2	31.6	0	0	26.9	0	0	72.7	0	0	36	0	0
S3	26.3	0	0	26.9	0	0	54.5	0	0	30.7	0	0
S4	5.26	0	0	15.4	0	0	36.4	0	0	13.3	0	0
S5	34.2	0	0	23.1	0	0	36.4	0	0	30.7	0	0
S6	13.2	0	0	15.4	0	3.85	0	0	0	12	0	1.45
S7	42.1	2.78	15.2	69.2	0	34.6	90.9	0	30	58.7	1.37	24.6
S8	13.2	0	0	15.4	3.85	0	18.2	0	0	14.7	1.37	0
S9	28.9	0	0	26.9	0	0	45.5	0	0	30.7	0	0
S10	15.8	0	0	15.4	0	0	18.2	0	0	16	0	0
S11	0	0	0	7.69	0	7.69	18.2	0	0	5.33	0	2.9
M1	18.4	0	0	38.5	0	0	54.5	0	0	30.7	0	0
N1	28.9	0	0	30.8	3.85	0	54.5	0	0	33.3	1.37	0
N2	42.1	0	0	53.8	0	0	63.6	0	0	49.3	0	0
NSP1	36.8	0	0	15.4	0	0	36.4	0	0	29.3	0	0
NSP2	7.89	0	0	11.5	0	0	27.3	0	0	12	0	0
NSP3	2.63	0	ND	11.5	0	ND	9.09	0	ND	6.67	0	ND
NSP4	2.63	0	ND	3.85	0	ND	0	0	ND	2.67	0	ND
NSP5	31.6	0	ND	34.6	0	ND	45.5	0	ND	34.7	0	ND
ORF3b	2.63	0	ND	3.85	0	ND	18.2	0	ND	5.33	0	ND
ORF8	7.89	0	ND	7.69	0	ND	18.2	0	ND	9.33	0	ND
ORF8_1	0	0	ND	0	0	ND	0	0	ND	0	0	ND
ORF8_2	5.26	0	ND	15.4	0	ND	27.3	0	ND	12	0	ND

IgG antibodies against the S_2sub_, S_FL_, S_FL3_ and N_FL_ were found in > 97.3% of the patients thus showing that these are the most suitable antigens for the production of serological tests for epidemiological studies. IgGs against the S_RBD_, S_1sub_ M_FL_ were also detected in 100% of the patients with severe and moderate disease, while the frequencies were ranging from 84 to 92% in patients with mild disease. From the individual predicted epitopes, S7—a 32 aa. peptide (V551-L582) located in the SD1 subdomain of the RBD, showed the highest prevalence of IgG responses (58.7%) thus suggesting that this is the immunodominant B cell epitope within the RBD.

The frequency of IgA responses against S_FL3_, S_FL_, S_RBD_, S_S1sub_, S_S2sub_, M_FL_ and N_FL_ ranged from 24.7 to 35.6% and all the responses except for N_FL_ showed the highest frequencies in patients with severe disease, followed by moderate and mild disease. IgAs against the predicted epitopes or other protein fragments were detected in a few cases, presumably because the antibody levels to monovalent antigens didn’t reach the limit of detection.

IgE antibodies were detected against N_FL,_ S_FL3_, S_FL_, S_RBD_, S_S1sub_, S_S2sub_, M_FL_ and S6, S7 and S11 epitopes, with N_FL_ being the most frequently recognized antigen. Anti-N_FL_ IgEs were detected in 100, 96.2 and 66.7% of patients with severe disease, moderate and mild disease, respectively. However, the IgEs were detectable only at serum dilution 1:5, hence the signal intensities and frequencies are not directly comparable with those of IgG and IgA.

### Correlation of antibody levels with severity of the disease

**Fig. 3:**
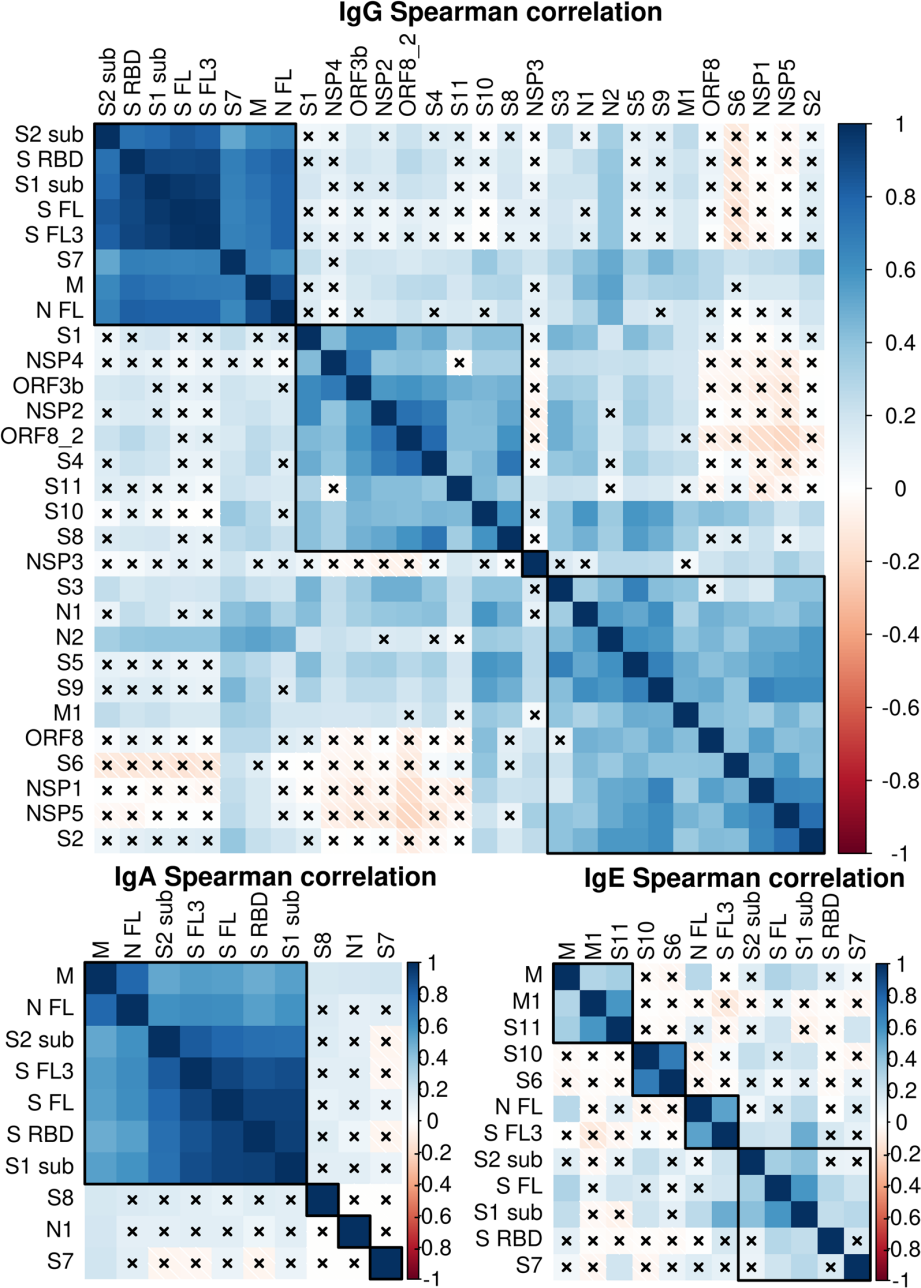
Spearman correlation analysis of the IgG, IgA and IgE responses against various SARS-CoV-2 antigens. The two tailed correlation analysis and hierarchical clustering was performed using R package. × insufficient number of samples with values > 0

To assess to what extent the antibody responses to various antigens are correlated, we performed Spearman correlation analysis (Fig. 3). This analysis revealed a cluster of strongly correlated IgG responses: anti-S_2sub_, S_RBD_, S_1sub,_ S_FL_, S_FL3_, S7, M and N_FL_ (Spearman *R* > 0.52; *P* < 2.4 × 10^–11^), Similarly, IgA responses to M_,_ N_FL,_ S_2sub,_ S_FL3_, S_FL_, S_RBD_ and S_1sub_ were highly correlated (Spearman *R* > 0.49; *P* < 2.7 × 10^–10^). IgE responses to S_1sub_ were correlated to S_2sub_ and S_FL_ (Spearman *R* > 0.33; *P* < 2.2 × 10^–4^) and anti-N_FL_ to S_FL3_ (Spearman *R* = 0.53; *P* = 4.5 × 10^–10^). We did not observe any significant negative correlation that would have suggested that the antibody responses to different antigens are mounted in a mutually exclusive manner.

**Fig. 4:**
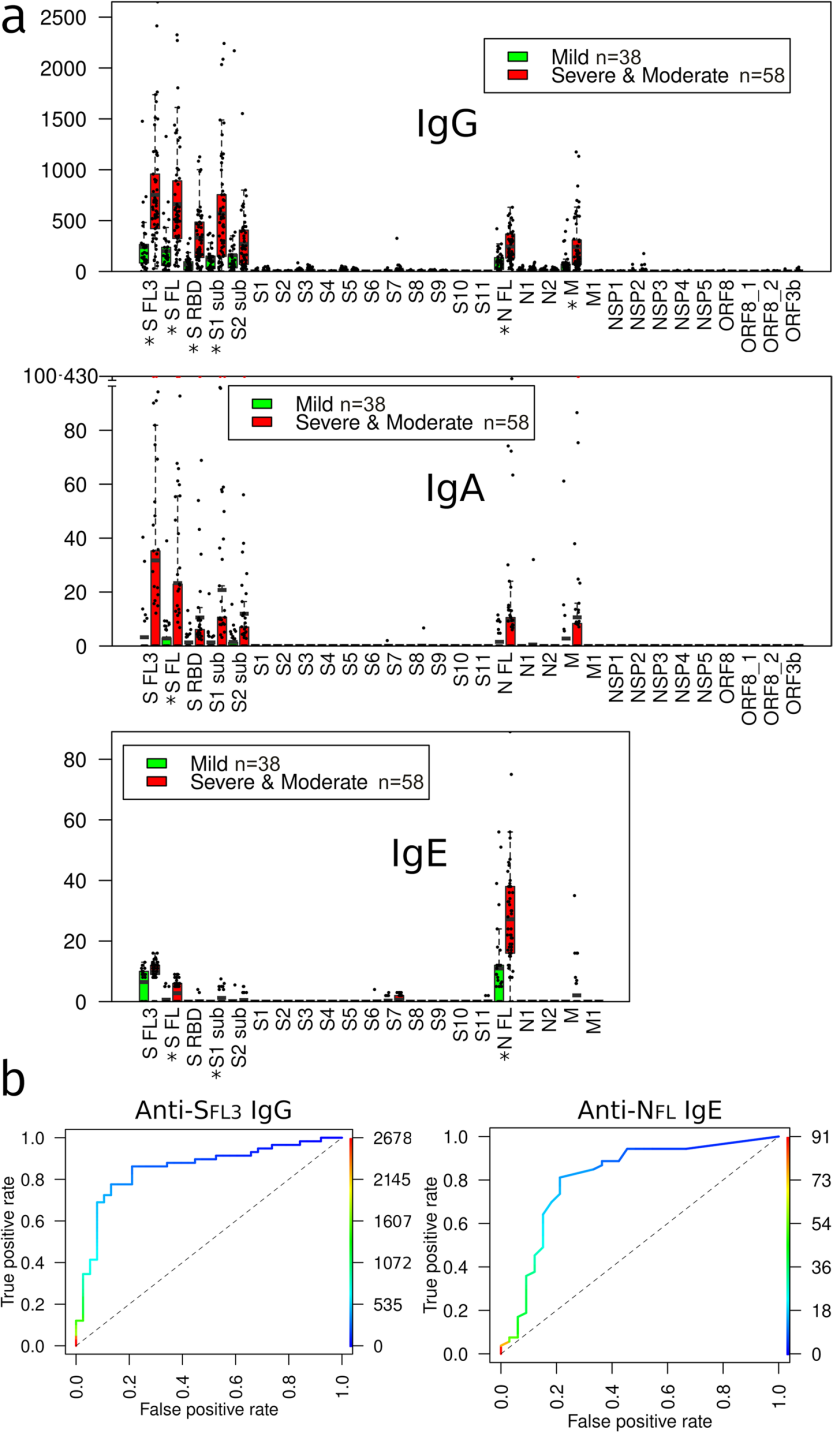
Correlation of the antibody levels with severity of the disease at > 14 DPS. **a** IgG, IgA and IgE antibody levels against each of the SARS-CoV-2 antigens in the groups of patients with severe/moderate and mild disease. Box plots show mean and 25th and 75th percentile, upper whisker—Q3 + 1.5 IQR, lower whisker—Q1−1.5 IQR; dots represent individual samples. * Adj. *P* < 0.05. **b** ROC curves showing the discrimination between patients with severe/moderate *vs* mild disease based on anti-S_FL3_ IgG and anti-N_FL_ IgE levels

Next, we assessed whether the antibody levels during the SARS-CoV-2 infection correlate with the disease severity. The mean levels of IgG, IgA and IgE antibodies against each antigen were compared between the groups of patients with severe and moderate *vs* mild disease in the samples collected > 14 DPS (Fig. 4a). The samples from severe and moderate groups were combined due to too small number of cases in the severe group that precluded the statistical analysis. For patients who had several sequential samples, the sample with the highest reactivity was included. This analysis revealed that IgG levels against the N_FL_, S_RBD_, S_FL3_, S_1sub_ and S_FL_ were significantly higher in patients with severe/moderate disease as compared with those with mild disease. Likewise, anti-S_FL_ IgA and anti-S_1sub_, N_FL_ and S_FL_ IgE levels were significantly higher in the severe/moderate disease group (all adj. *P* < 0.05). ROC curve analysis showed that anti-S_FL3_ IgGs could distinguish patients with severe/moderate disease from those with mild disease with AUC of 0.851 (*P* = 6.8 × 10^–9^), whereas anti-NFL IgE had an AUC of 0.809 (*P* = 1.6 × 10^–6^) (Fig. 4b).

**Fig. 5:**
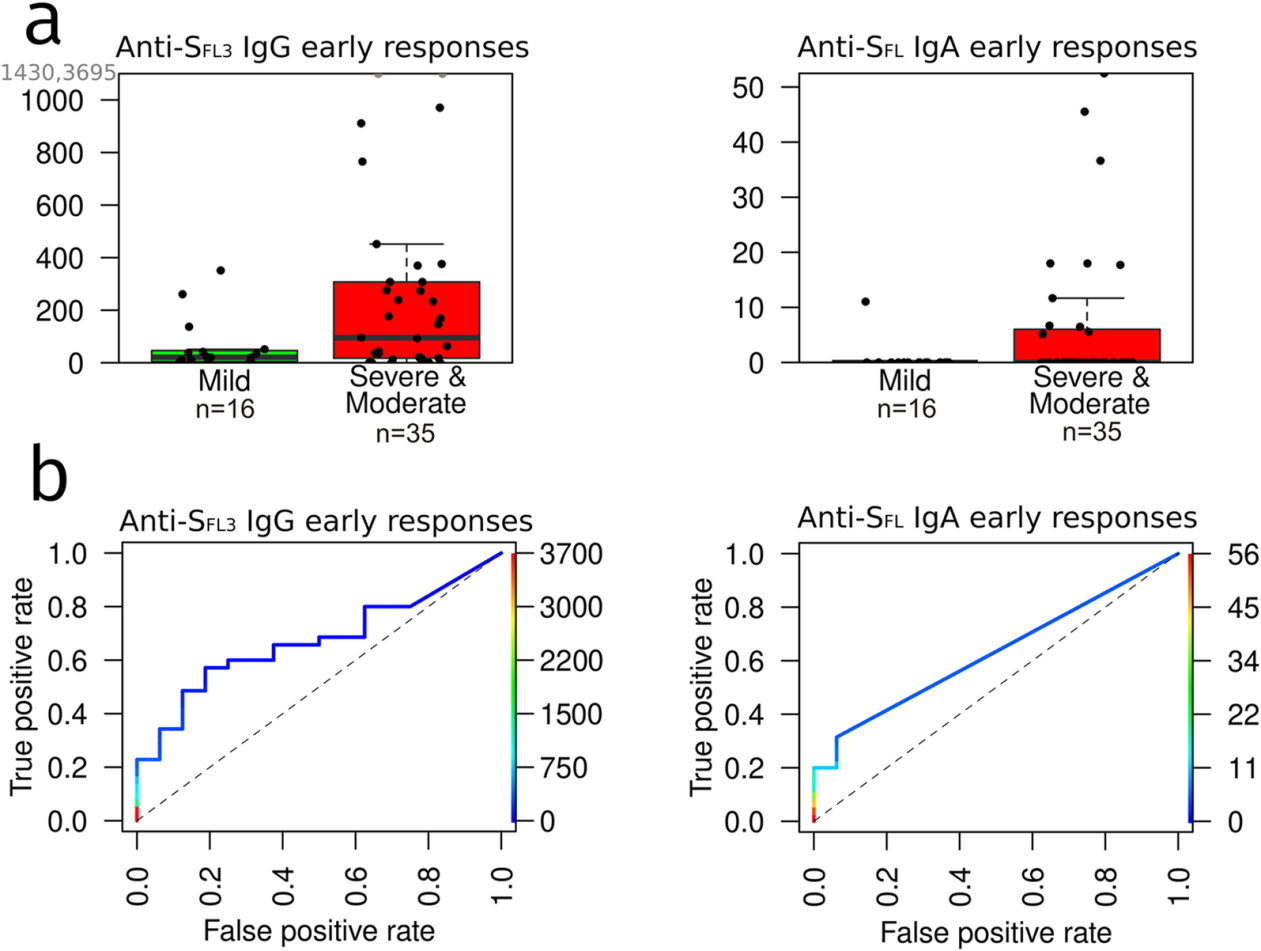
Correlation of the early antibody levels (3–14 DPS) with the disease severity. **a** Anti-S_FL3_ IgG and anti-S_FL_ IgA levels in the groups of patients with severe/moderate (n = 35) vs mild disease (n = 16). Box plots show median and 25th and 75th percentile, upper whisker—Q3 + 1.5 IQR, lower whisker—Q1−1.5 IQR; dots represent individual samples. **b** ROC curves showing the discrimination between patients with severe/moderate *vs* mild disease based on early anti-S_FL3_ IgG and anti-S_FL_ IgA levels

Next, the levels of antibodies against the most reactive antigens were compared between patients with moderate/severe *vs* mild disease in samples collected at 3–14 DPS. Results revealed that anti-S_FL3_ IgG and anti-S_FL_ IgA levels were significantly higher in patients with moderate/severe disease than with mild disease (*P* < 0.05) (Fig. 5a). However, ROC curve analysis showed that these markers could distinguish between these groups of patients with a moderate AUC of 0.67 (*P* = 0.05) and 0.629 (*P* = 0.05), respectively (Fig. 5b).

## Discussion

Most of the studies investigating antibody response in COVID-19 patients have measured antibodies against full-length S or N proteins or their domains and have primarily focused on IgM and IgG classes. Here we report the profiling of antibody responses against multiple SARS-CoV-2 antigens or their fragments using a 30-antigen array and correlation of IgM, IgG, IgA and IgE antibody levels with the disease severity. Given that many of the predicted epitopes within SARS-CoV-2 proteome have been shown to have homology to human proteins [14] and patients with severe disease have been shown to develop autoantibodies against type I interferons [15] and various other immunomodulatory proteins [16], we initially hypothesized that in patients with severe disease, the antibody response might be skewed towards epitopes that share homology to human proteins and hence may lead to pathogenic priming and induction of autoimmunity, whereas in patients with mild disease, the antibody response would be directed mainly against S_RBD_, S_1sub_ and S_2sub_ that have been shown to neutralize the viral infection [2, 3]. Our results, however, did not support such a hypothesis.

Instead, we observed that antibody responses against various antigens are highly correlated and we failed to identify specific epitopes that were preferentially recognized by sera from patients with severe or mild disease. The peak levels of IgG, IgA and IgE responses to various forms of S, N and M proteins were higher in patients with severe/moderate disease than in patients with mild disease. This finding may seem paradoxical, however, similar results have been reported in several other studies. Chen et al. showed that IgG levels against the RBD, S1 and S2 subunits of S protein were positively correlated to disease severity and demonstrated that anti-RBD antibodies have a neutralizing capacity [2]. Similarly, Lynch et al. reported that the IgG and IgM levels against the RBD and N proteins were significantly higher in patients admitted to the intensive care units compared to those with a milder disease in all time intervals [17]. Li et al. explored the IgG responses against various structural and non-structural SARS-CoV-2 antigens and found that the positivity rate and/or IgG levels against S1, NSP1, NSP7, NSP8, RdRp, ORF3b and ORF9b were significantly higher in patients with severe than non-severe disease [18]. From these non-structural proteins, only NSP1 and ORF3b were represented on our antigen array. In line with this study, we found that the positivity rate for anti-ORF3b IgGs was correlated with the severity of disease, whereas we did not find a significant correlation between anti-NSP1 IgG response and the severity of disease. However, the overall anti-NSP1 IgG positivity rate was also lower in our study (29.3% vs 38%) thus suggesting that our assay had lower sensitivity for detecting anti-NSP1 IgGs. In addition, we found that the positivity rates of IgGs against NSP2 and NSP5 were higher in patients with severe disease, however we do not recon them as clinically useful markers because the positivity rates even in patients with severe disease were below 50%. Two other studies reported rapid and stronger antibody responses at an early stage of the disease in patients with severe/moderate disease but no significant differences in the peak levels at subsequent time points [12, 19]. These differences most likely are related to the sensitivity and linear dynamic range of the assays, definition of the severity of the disease and the statistical power of the study.

These findings suggest that antibodies against the SARS-CoV-2 may have dual roles in the COVID-19 disease. On one hand, it has been demonstrated that antibodies against the RBD, S1 and S2 subunits can neutralize the virus [2, 3] and possibly attenuate the viral replication and reinfection [20]. However, the fact that patients with mild disease have low levels of antibodies, including the neutralizing anti-RBD IgGs, suggests that antibodies are not essential for the elimination of the virus and supports the idea that CD8^+^T cells are the main players in the protective immune response against the SARS-CoV-2 infection [21, 22]. Unfortunately, we did not have an opportunity to test the viral load in our cohort of patients, therefore we cannot assess whether the high antibody levels are caused by higher viral loads. However, several studies have suggested that the viral load is not correlated with the severity of the disease and/or anti-N and anti-S_RBD_ IgG and IgM levels [23, 24]. Therefore, we hypothesize that antibodies may also exacerbate the severity of the disease via antibody-dependent enhancement (ADE). Generally, ADE may occur through two distinct mechanisms: antibody-mediated virus uptake into macrophages or other FcγRIIa-expressing phagocytic cells leading to increased viral replication and spread, and excessive Fc-mediated effector functions or immune complex formation causing hyperinflammation [25]. There is no evidence that SARS-CoV-2 infects macrophages [26], however it might be possible that antibodies contribute the hyperinflammation, for example, by stimulating the production of pro-inflammatory cytokines.

This is the first study reporting that SARS-CoV-2 elicits IgE responses against N, S and M proteins in sera of COVID-19 patients and their levels positively correlate with the disease severity. IgE is a classical mast cell activator. The binding of IgE-coated antigen to FcεRI receptors on mast cells results in the crosslinking of the IgE-FcεRI complex that in turn triggers mast cell activation and release of histamine and various pro-inflammatory chemokines and cytokines including IL-6, IL-1β and TNF-α [27, 28]. Several studies have shown that mast cells are activated in severe COVID-19 patients and suggested that mast cell activation syndrome may be implicated in the development of cytokine storm and hyperinflammation [28–30]. Hence, this study suggests a link between elevated antibody levels in severe COVID-19 and mast cell activation and supports the idea of using IgE blocking drugs such as omalizumab for the treatment of COVID-19 [31].

Furthermore, our data suggest that high levels of anti-SARS-CoV-2 antibodies may be used as a biomarker for predicting the disease severity. However, such a biomarker would be clinically meaningful only if detectable at an early phase of the disease. Our patient cohort was not suitable for defining the day of seroconversion in each patient, therefore we couldn’t assess on which day after the onset of symptoms the differences in the antibody levels between patients with severe/moderate and mild disease become significant. The analysis of samples taken from 3 to 14 DPS showed that the best of the antibody biomarkers—anti-S_FL3_ IgG and anti-S_FL_ IgA levels, had only moderate AUCs of 0.67 and 0.629, respectively, and combining different antibody biomarkers did not improve the prognostic performance. Hence, the early antibody responses may serve as an additional biomarker for identifying the patients at risk of developing severe disease, however, the prognostic value is not sufficiently high to recommend it as an independent prognostic factor.

Moreover, the antigen array developed in this study can be exploited for distinguishing vaccine-induced antibody responses from virus-induced antibody responses that can be of high importance for epidemiological studies, assessing vaccine efficacy and resolving specific clinical cases.

## Conclusions

This study revealed that antibody responses to various SARS-CoV-2 antigens are highly correlated and their levels are significantly higher in patients with severe/moderate disease than in patients with mild disease. Moreover, our study shows for the first time that SARS-CoV-2 elicits serum IgE responses that may provide a missing link between elevated antibody levels and mast cell activation. Hence, our data provide important insights into the pathogenesis of COVID-19 and have implications in planning and interpreting antibody-based epidemiological studies.

## Supplementary Information

**Additional file 1: Table S1.** Predicted SARS-CoV-2 epitopes expressed in-house. **Table S2.** Commercial SARS-CoV-2 proteins and human control proteins.

**Additional file 2: Fig. S1.** Performance of the SARS-CoV-2 antigen array. **a** A representative image of testing anti-SARS-CoV-2 IgG antibodies in serum from a COVID-19 patient. **b** Dynamic range of the IgG assay. The antigen array was tested with serial dilutions of a serum sample from a COVID-19 patient. Each dilution was tested in duplicates.

## Data Availability

The datasets used and/or analyzed during the current study are available from the corresponding author on reasonable request.

## Abbreviations

ADE: Antibody-dependent enhancement
AUC: Area under the curve
COVID-19: Coronavirus disease 2019
DPS: Days after the onset of symptoms
RBD: Receptor-binding domain
ROC curve: Receiver operating characteristic curve
SARS-CoV-2: Severe acute respiratory syndrome coronavirus 2
