# Supplementary figures and images for: Early and strong antibody responses to SARS-CoV-2 predict disease severity in COVID-19 patients
Source: J Transl Med. 2022 Apr 15;20:176. doi: 10.1186/s12967-022-03382-y (PMC9012069; doi:10.1186/s12967-022-03382-y)

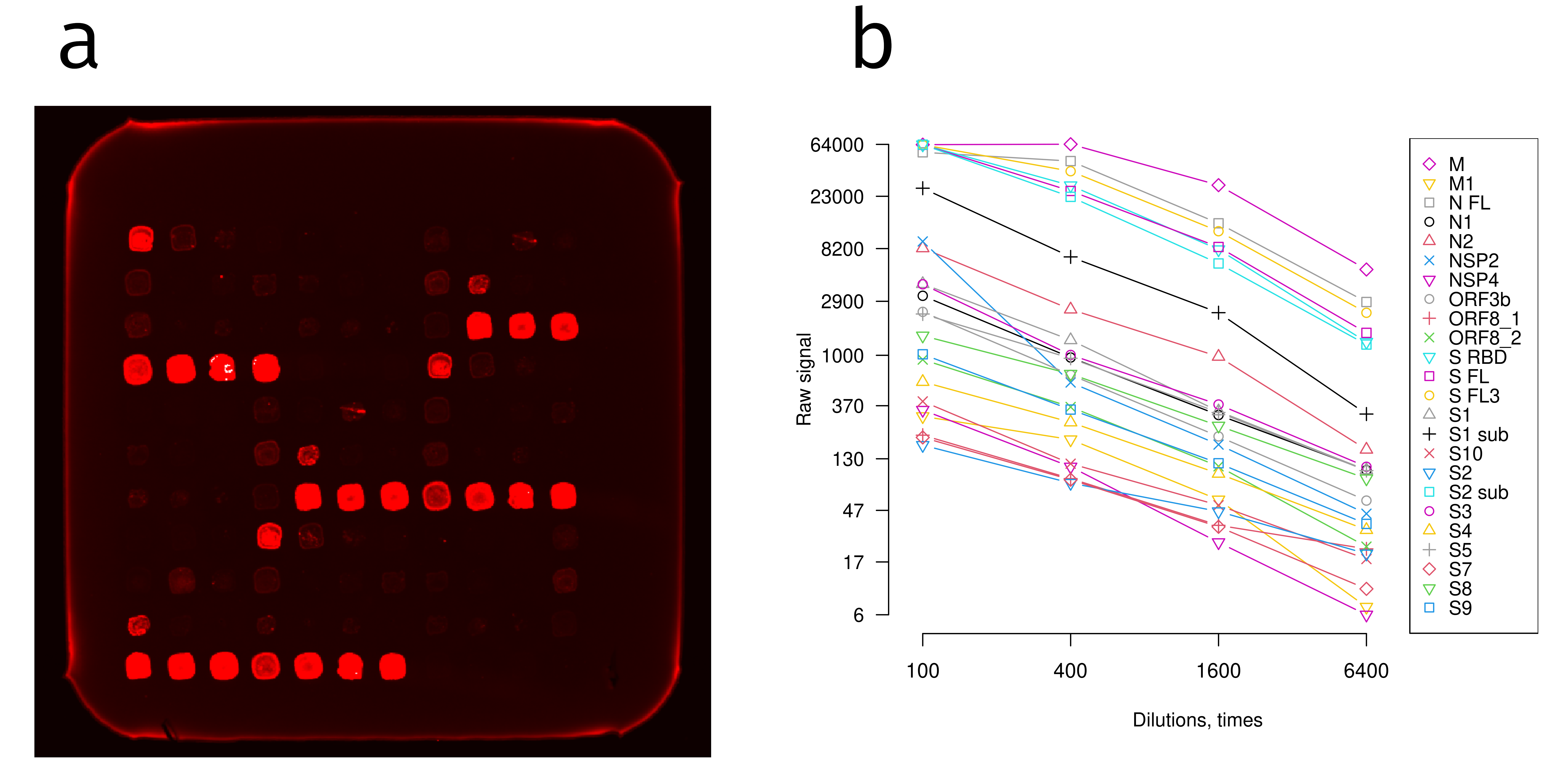

Supplement: Supplementary file 2 — Additional file 2: Fig. S1. Performance of the SARS-CoV-2 antigen array. a A representative image of testing anti-SARS-CoV-2 IgG antibodies in serum from a COVID-19 patient. b Dynamic range of the IgG assay. The antigen array was tested with serial dilutions of a serum sample from a COVID-19 patient. Each dilution was tested in duplicates. [file 12967_2022_3382_MOESM2_ESM.tiff]
